# Analysis of potential immune-related genes involved in the pathogenesis of ischemia-reperfusion injury following liver transplantation

**DOI:** 10.3389/fimmu.2023.1126497

**Published:** 2023-03-16

**Authors:** Jiayu Guo, Shangting Han, Qi Chen, Tianyu Wang, Bo Yu, Jiangqiao Zhou, Tao Qiu

**Affiliations:** ^1^ Department of Organ Transplantation, Renmin Hospital of Wuhan University, Wuhan, Hubei, China; ^2^ Department of Urology, Renmin Hospital of Wuhan University, Wuhan, Hubei, China

**Keywords:** liver transplantation, ischemia-reperfusion injury, immune, differentially expressed genes, hub genes

## Abstract

**Background:**

Hepatic ischemia-reperfusion (I/R) injury is an unavoidable pathological process that occurs after liver transplantation. However, the immune-related molecular mechanism still remains unclear. This study aims to further explore the biological mechanisms of immune-related genes in hepatic I/R injury.

**Methods:**

Gene microarray data was downloaded from the Gene Expression Omnibus (GEO) expression profile database and the differentially expressed genes (DEGs) were taken for intersection. After identifying common DEGs, functional annotation, protein-protein interaction (PPI) network, and modular construction were performed. The immune-related hub genes were obtained, which their upstream transcription factors and non-RNAs were predicted. Validation of the hub genes expression and immune infiltration were performed in a mouse model of hepatic I/R injury.

**Results:**

A total of 71 common DEGs were obtained from three datasets (GSE12720, GSE14951, GSE15480). The GO and KEGG enrichment analysis results indicated that immune and inflammatory response played an important role in hepatic I/R injury. Finally, 9 immune-related hub genes were identified by intersecting cytoHubba with immune-related genes, including SOCS3, JUND, CCL4, NFKBIA, CXCL8, ICAM1, IRF1, TNFAIP3, and JUN.

**Conclusion:**

Our study revealed the importance of the immune and inflammatory response in I/R injury following liver transplantation and provided new insights into the therapeutic of hepatic I/R injury.

## Introduction

Hepatic ischemia-reperfusion (I/R) injury is a common and unavoidable complication of surgical procedures such as liver transplantation and liver resection (1), and is a primary cause of graft dysfunction and post-transplantation hepatic failure (2). The shortage of donor organs has forced expanded criteria for grafts resulting in a higher probability of hepatic I/R injury (3) worsening both short- and long-term outcomes of transplantation and limiting patient survival. Thus, there is a critical need to explore the molecular mechanisms of hepatic I/R injury to improve the challenges faced.

Hepatic I/R injury consists of two phases, ischemic insult and inflammation-mediated reperfusion injury. During the ischemic phase, tissue hypoxia, ATP depletion and pH changes due to interrupted blood flow leads to hepatocyte damage or death (4). Subsequently, restoration of blood flow disrupts normal liver metabolism, triggering a series of inflammatory cascades and damage-associated molecular patterns that exacerbate liver cell damage (5). Organ-specific inflammation caused by activation of the host innate immune response is central to this process (6, 7). The broad regulatory patterns and mechanisms of immune-related genes involved in hepatic I/R injury remain largely unknown, and further research is needed.

Bioinformatics is widely used to analyze cancer genome sequencing data and obtain biologically meaningful information (8). The current study used data mining and analysis techniques to differentially analyze pre-ischemic and post-reperfusion liver transplant tissue specimens and obtain differentially expressed genes (DEGs). Comprehensive bioinformatics and enrichment analysis was used to idenrify common DEGs and their functions in hepatic I/R injury. In addition, the STRING database and Cytoscape software were used to build PPI networks, perform gene module analysis and identify hub gene. Finally, we identified nine immune-related essential hub genes and further analyzed the transcription factors and upstream miRNAs and LncRNAs of these genes, and validated their expression. An I/R mouse model was also developed and validated. This study provides new insights into the biological mechanisms underlying the role of hub immune-related genes in hepatic I/R injury.

## Materials and methods

### Data source

Related gene expression datasets were identified using “liver transplantation” MeSH Terms AND “Homo sapiens” Organism AND Expression profiling *via* GEO (http://www.ncbi.nlm.nih.gov/geo) (9), a public database containing a large number of high-throughput sequencing and microarray datasets submitted by research institutions worldwide. The GSE12720 dataset contains 13 liver tissues from pre-ischemic donors and 13 liver tissues from reperfused livers after liver transplantation. The GSE14951 dataset contains five liver tissues from pre-ischemic donors and five liver tissues from reperfused livers after liver transplantation. The GSE15480 dataset contains six liver tissues from pre-ischemic donors and six liver tissues from reperfused livers after liver transplantation. Details of three datasets are shown in **Table 1**. The probe names were transformed into gene symbols based on platform annotation information. Moreover, immune-related genes were obtained from the Immunology Database and Analysis Portal (IMMPORT) database (http://www.immport.org/) (10).

**Table 1:** Details of a transcriptomic dataset of liver transplant patients.

GEO accession	Platform	Sample organism	Pre-ischemic sample (n)	Post-reperfusion sample (n)	Time of reperfusion (h)	Contributors (Year)
GSE12720	GPL570	*Homo sapiens*	13	13	1	Kurian SM et al. (2019)
GSE14951	GPL570	*Homo sapiens*	5	5	2-3	Conti A et al. (2019)
GSE15480	GPL6244	*Homo sapiens*	6	6	1.5	Raza A et al. (2018)

### Differential expression analysis

The three raw datasets were downloaded using the R “GEOquery” package, and then performed gene annotation, gene filtering, normalization, etc. on the three datasets. The differentially expressed genes (DEGs) were analyzed and compared between the pre-ischemic and reperfused liver tissue controls using the R “limma” package. Only those genes with adjusted P-value < 0.05 and |log2FC (fold change)| ≥ 0.5 were considered DEGs. The R “ggplot2” package was used to draw volcano plots and the R “pheatmap” package was used to draw heatmaps for data visualization.

### Venn method

The venn diagram was used to analyze common DEGs. Intersections of the DEGs from the three datasets, as well as the intersection between hub genes obtained from CytoHubba and immune-related genes were identified using the Venny online tool, version 2.1 (https://bioinfogp.cnb.csic.es/tools/venny/index.html).

### Functional enrichment and protein–protein interaction network construction

In order to explore the functions of common DEGs between the three datasets and the involved modular genes obtained using molecular complex detection technology (MCODE), gene ontology (GO) and Kyoto Encyclopedia of Genes and Genomes (KEGG) enrichment analyses were conducted using the R “org.Hs.eg.db” and “ClusterProfiler” packages. P < 0.05 was considered statistically significant. The R “ggplot2” package was used to visualize the enrichment results.

The STRING database (https://string-db.org/) (11) was used to identify the relationships between DEGs that were present in all three databases, including direct binding relationships, or coexisting upstream and downstream regulatory pathways, and a PPI network with complex regulatory relationships was created. Interactions with a combined score of > 0.4 were considered statistically significant. The nodes represented genes, and the edges represented the links between the genes. Cytoscape software (https://cytoscape.org) (12) was used to develop the PPI network.

### Module analysis

Cytoscape’s plug-in molecular complex detection technology (MCODE) (13) was used to analyze key functional modules. The function of MCODE is to select key sub-networks, i.e., modules. The selection criteria were set as: K-core = 2, degree cutoff = 2, max depth = 100, and node score cutoff = 0.2. GO and KEGG analyses of the involved modular genes were then conducted using the R “org.Hs.eg.db” and “ClusterProfiler” packages.

### Selection and analysis of hub genes

The hub genes were identified using the cytoHubba plug-in of Cytoscape. TheDegree algorithm was used to evaluate and select hub genes. And immune-related hub genes as candidates for subsequent analysis. A co-expression network of the immune-related hub genes was conducted using GeneMANIA (http://www.genemania.org/) (14), a reliable tool for identifying internal associations in gene sets.

### Prediction of transcription factors

Transcriptional Regulatory Relationships Unraveled by Sentence-based Text mining (TRRUST) (15) is a database used to predict transcriptional regulatory networks, which contain the target genes corresponding to TFs and the regulatory relationships between TFs. The TRRUST database includes 8,444 and 6,552 TFs target regulatory relationships from 800 human TFs and 828 mouse TFs, respectively. TRRUST database was used to obtain the TFs that regulate immune-related hub genes, and an adjusted P-value < 0.05 was considered significant.

### Prediction of gene-miRNA and miRNA-LncRNA interactions

Potential immune-related hub genes targeted miRNAs were predicted using the TargetScan (http://www.targetscan.org/vert_72/) (16), miRTarBase (https://mirtarbase.cuhk.edu.cn/php/index.php) (17), and miTDB (http://mirdb.org/) (18) databases. The miRNAs for each immune-related hub gene predicted by the three databases were taken to intersect. The results obtained from the intersection were further processed usingCytoscape, miRNAs targeting more than two genes were selected. The upstream LncRNA of the obtained miRNAs was predicted using StarBase v2.0 (19), and the interactions were visualized using Cytoscape

### Animals and treatment

Twenty 6-8 week-old male C57BL/6J mice weighing 22–25 g were acquired from the Center of Experimental Animals at the Renmin hospital of Wuhan University, Hubei, China. The animal experiments were conducted according to the Guide for the Care and Use of Laboratory Animals, and the study protocol was approved by the Laboratory Animal Welfare and Ethics Committee of the Renmin hospital of Wuhan University. Mice were kept in an air-filtered, temperature-controlled (22–24°C), humidity-controlled (40-70%), and light-controlled room and were permitted free access to a standard diet.

The stable mouse model of partial liver warm ischemia was constructed according to the classical method, as previously described. The mice were anaesthetized with 3% pentobarbital sodium prior to surgery. Sham-operated mice underwent the same surgical procedure without clamping of the blood vessels. The 70% liver tissue ischemia model, in which the portal vein and hepatic artery of the middle and left lobes areblocked with non-invasive vascular clamps, was used during surgery. Ischemia was performed for 60 minutes, and reperfusion times varied. Animals were euthanized post-procedure, and the serum and liver samples were immediately collected for further analyses.

### Analysis of liver function

The concentrations of serum alanine aminotransferase (ALT) and serum aspartate aminotransferase (AST) were measured using the ADVIA 2400 Biochemistry Analyzer (Siemens, Tarrytown, NY, USA).

### Hematoxylin-eosin staining

After fixation with 4% paraformaldehyde, liver samples were embedded in paraffin and cut into 5-μm sections. H&E staining was used to assess histopathological liver damage and quantified using Image J software. The percentage of necrotic area in the total area of the tissue section was quantified in more than five fields for each mouse.

### Terminal deoxynucleotidyl transferase dUTP nick end labeling

An apoptosis assay kit (ApopTag^®^ Plus *In Situ* Apoptosis Fluorescein Detection Kit, S7111; Millipore, USA) was used to rapidly detect apoptosis induced by I/R injury. TUNEL assay was performed according to the manufacturer’s instructions. Images were obtained using fluorescent microscopy. Quantification of TUNEL-positive cells in each field of the tissue section was performed using Image J software

### Immunohistochemistry and immunofluorescence staining

Immunohistochemical and immunofluorescence staining was conducted to analyze the protein expression levels of iNOS, CD11b, and CD206. All procedures were performed according to the manufacturer’s recommendations. Positive areas were compared between groups using microscopy and images were analyzed with Image J software.

### Quantitative real-time PCR

The total mRNA was extracted from cultured cells using TRIzol reagent (Invitrogen Life Technologies, Carlsbad, CA, United States). RNAs were reverse transcribed into cDNA using ImProm-II™ Reverse Transcription System (Promega, USA) according to the instructions of the manufacturer. Quantitative RT-PCR with cDNA as template and β-Actin as internal reference to analyze the relative mRNA expression of each gene. The primer sequences used for each gene have been listed in **Supplementary Table S1**. The 2^−ΔΔCT^ method was used to calculated the relative mRNA expression levels which were normalized against β-Actin.

### Statistical analysis

All data are expressed as the mean ± standard deviation. SPSS version 19.0 was used for statistical analysis. The student’s t-test was used to compare differences between groups. Differences were considered significant when the *P* value was <0.05. All experiments were performed at least three times.

## Results

### Identification of DEGs

**Figure 1:**
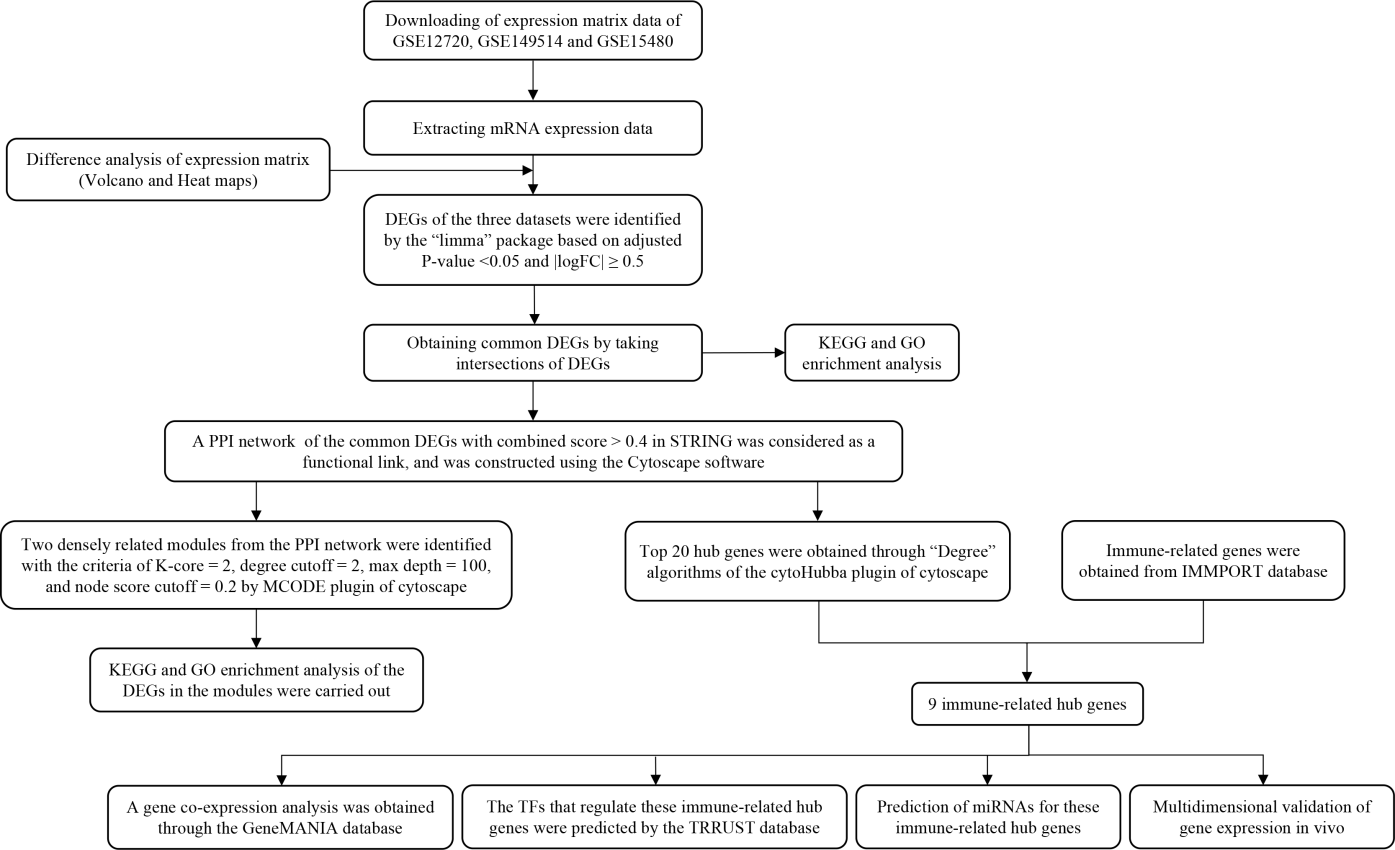
Schematic representation of the research process.

**Figure 2:**
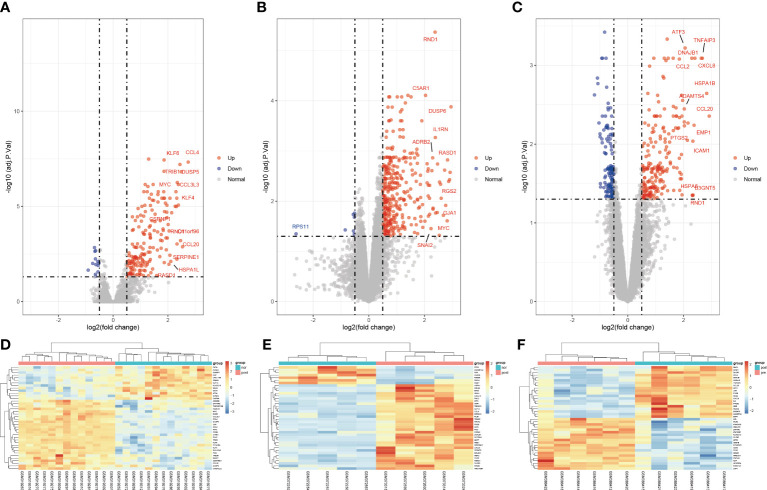
Volcano diagram and heat diagram. **(A–C)** Volcano map of GSE12720, GSE14951 and GSE15480. Upregulated genes are marked in light red; downregulated genes are marked in light blue. **(D–F)** Heat map of differentially expressed genes (DEGs) in GSE12720, GSE14951 and GSE15480. The top 40 DEGs for each dataset are shown on the heat map.

To clarify the process of this research, the research flowchart is shown in **Figure 1**. Original data were downloaded from the GSE12720, GSE14951 and GSE15480 datasets in the GEO database. After standardizing the microarray data, differential expression analysis was performed and DEGs were identified. In the GSE12720 dataset, the transcriptome data from 13 pre-ischemic donor liver tissues and 13 liver tissues after reperfusion were analyzed using criteria of |log2FC| ≥ 0.5 and P < 0.05, yielding 192 DEGs (179 upregulated and 13 downregulated). In the GSE14951 dataset, transcriptome data from five pre-ischemic donor liver tissues and five liver tissues after reperfusion were analyzed using criteria of |log2FC| ≥ 0.5 and P < 0.05, yielding 372 DEGs (365 upregulated and 7 downregulated). In the GSE15480 dataset, the transcriptome data from six pre-ischemic donor liver tissues and six liver tissues after reperfusion were analyzed using criteria of |log2FC| ≥ 0.5 and P < 0.05, yielding 353 DEGs (212 upregulated and 141 downregulated). The corresponding heatmaps and volcano plots are shown in **Figure 2**. Details of the three datasets are presented in **Table 1**.

### Function enrichment analysis of common DEGs

**Figure 3:**
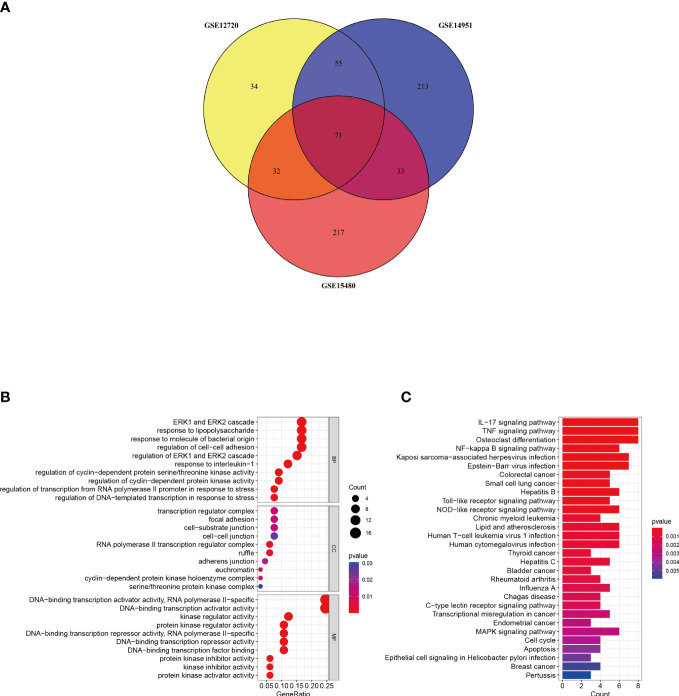
Identification and analysis of DEGs. **(A)** The three datasets show an overlap of 71 DEGs on the Venn diagram. **(B, C)** GO and KEGG enrichment analysis results of common DEGs. GO, gene ontology; and KEGG, Kyoto Encyclopedia of Genes and Genomes. p < 0.05 was considered significant.

A Venn diagram was created to determine the intersection between the DEGs from the three datasets and 71 common DEGs were identified (**Figure 3A**). To analyze the biological functions and involed pathways, GO and KEGG enrichment analysis were conducted. GO analysis showed that these genes were mostly enriched in the ERK1 and ERK2 cascade (p = 1.95E-08), response to lipopolysaccharide (p = 2.90E-08), response to interleukin-1 (p = 3.76E-08), regulation of transcription from the RNA polymerase II promoter in response to stress (p = 6.38E-07), and DNA-binding transcription activator activity (p = 3.40E-12) (**Figure 3B**). KEGG pathway enrichment analysis showed that the three significant enrichment pathways were IL-17 signaling pathway (p = 1.26E-08), TNF signaling pathway (p = 5.07E-08), NF-kappa B signaling pathway (p = 9.80E-06), Toll-like receptor signaling pathway (p = 1.3E-04), NOD-like receptor signaling pathway (p = 2.4E-04), and Human T-cell leukemia virus 1 infection (p = 6.6E-04) (**Figure 3C**). These results strongly indicated that immune response, inflammatory response and injury repair actions are involved in the development and occurrence of hepatic I/R injury.

### Construction of PPI network of DEGs and module analysis

**Figure 4:**
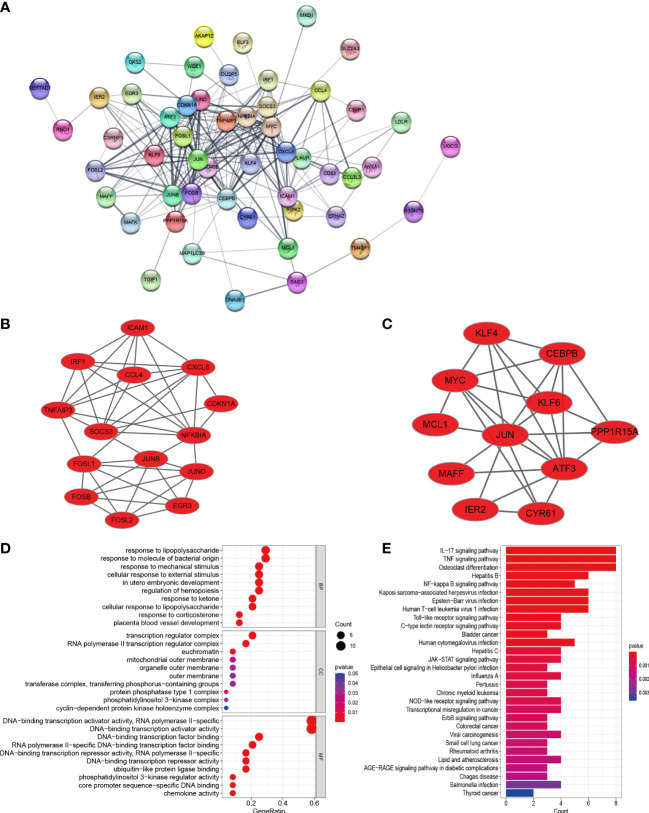
PPI network, significant gene module, and enrichment analysis of the modular genes. **(A)** Cytoscape network visualization of the 52 nodes and 218 edges based on the STRING online database with an interaction score of > 0.4. The nodes represent genes and the edges represent links between genes. Two key modules were identified by MCODE and used for network gene clustering identification. **(B)** Cluster 1. **(C)** Cluster 2. **(D, E)** GO and KEGG enrichment analysis results of the modular genes. p < 0.05 was considered significant.

In order to explore the mutual interaction between common DEGs, the PPI network of common DEGs with a combined score of > 0.4, which contained 52 nodes and 218 interaction pairs, was constructed using Cytoscape (**Figure 4A**). Two closely related gene modules were obtained by MCODE plug-in of Cytoscape, including 25 common DEGs and 73 pairs of interaction (**Figures 4B, C** and **Table 2**). The genes from the obtained modules were then subjected to functional enrichment analysis. GO analysis showed that these genes are associated with response to lipopolysaccharide, cellular response to external stimulus, transcriptional activation of DNA, and chemokine activity (**Figure 4D**). KEGG pathway analysis showed that these genes were primarily involved in the response to IL-17 signaling pathway, TNF signaling pathway, NF-kappa B signaling pathway, and NOD−like receptor signaling pathway (**Figure 4E**).

**Table 2:** Data downloaded from STRING were processed using molecular complex detection technology to further mine gene clusters.

Cluster	Score (Density*#Nodes)	Nodes	Edges	Node Names
1	6.769	14	44	ICAM1, JUNB, CXCL8, TNFAIP3, CCL4, IRF1, FOSL2, FOSL1, NFKBIA, FOSB, EGR3, JUND, CDKN1A, SOCS3
2	5.8	11	29	CEBPB, KLF6, KLF4, JUN, MYC, ATF3, CYR61, IER2, MAFF, PPP1R15A, MCL1

### Selection and analysis of hub genes

**Figure 5:**
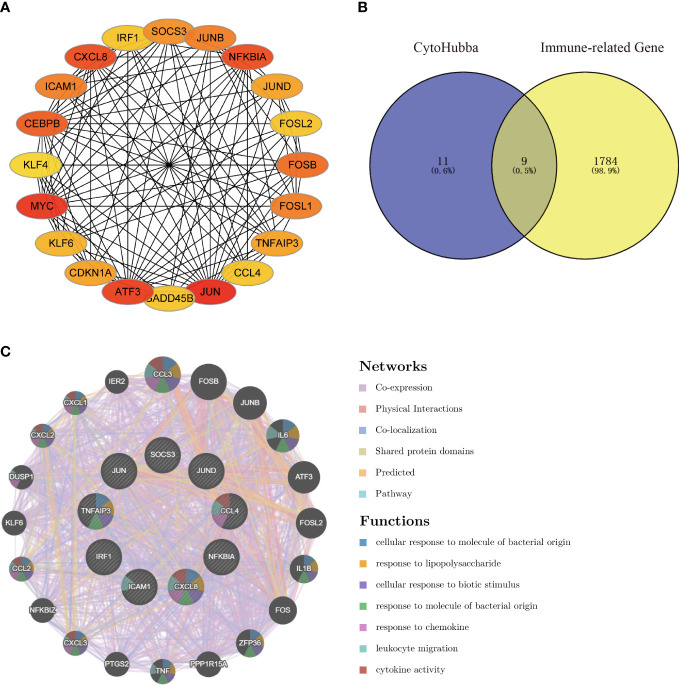
Venn diagram and co-expression network of the hub genes. **(A)** The top 20 genes were obtained by cytoHubba’s Degree algorithm and used them for network gene clustering identification. **(B)** Venn diagram of immune-related hub genes. **(C)** Immune-related hub genes and their co-expression genes were analyzed by GeneMANIA.

Using the Degree algorithm from the plug-in cytoHubba of Cytoscape, the top 20 hub genes (SOCS3, JUNB, NFKBIA, JUND, FOSL2, FOSB, FOSL1, TNFAIP3, CCL4, JUN, GADD45B, ATF3, CDKN1A, KLF6, MYC, KLF4, CEBPB, ICAM1, CXCL8, IRF1) were calculated (**Figure 5A**). To explore immune-associated hub genes involved in hepatic I/R injury, immune-associated genes were obtained from the IMMPORT database. Subsequently, the Venn method was used to analyze the intersection between hub genes obtained in cytoHubba and immune-associated genes.Nine overlapping immune-associated hub genes (SOCS3, JUND, CCL4, NFKBIA, CXCL8, ICAM1, IRF1, TNFAIP3, JUN) were identified (**Figure 5B**). Based on the Gene MANIA database, the co-expression network and associated functions of these genes were assessed. The genes had a complex PPI network with a 78.61% co-expression, 8.08% physical interaction rate, 6.42% co-localization rate, 1.95% predicted rate, and 0.35% pathway rate (**Figure 5C**).

### Prediction of TFs

**Figure 6:**
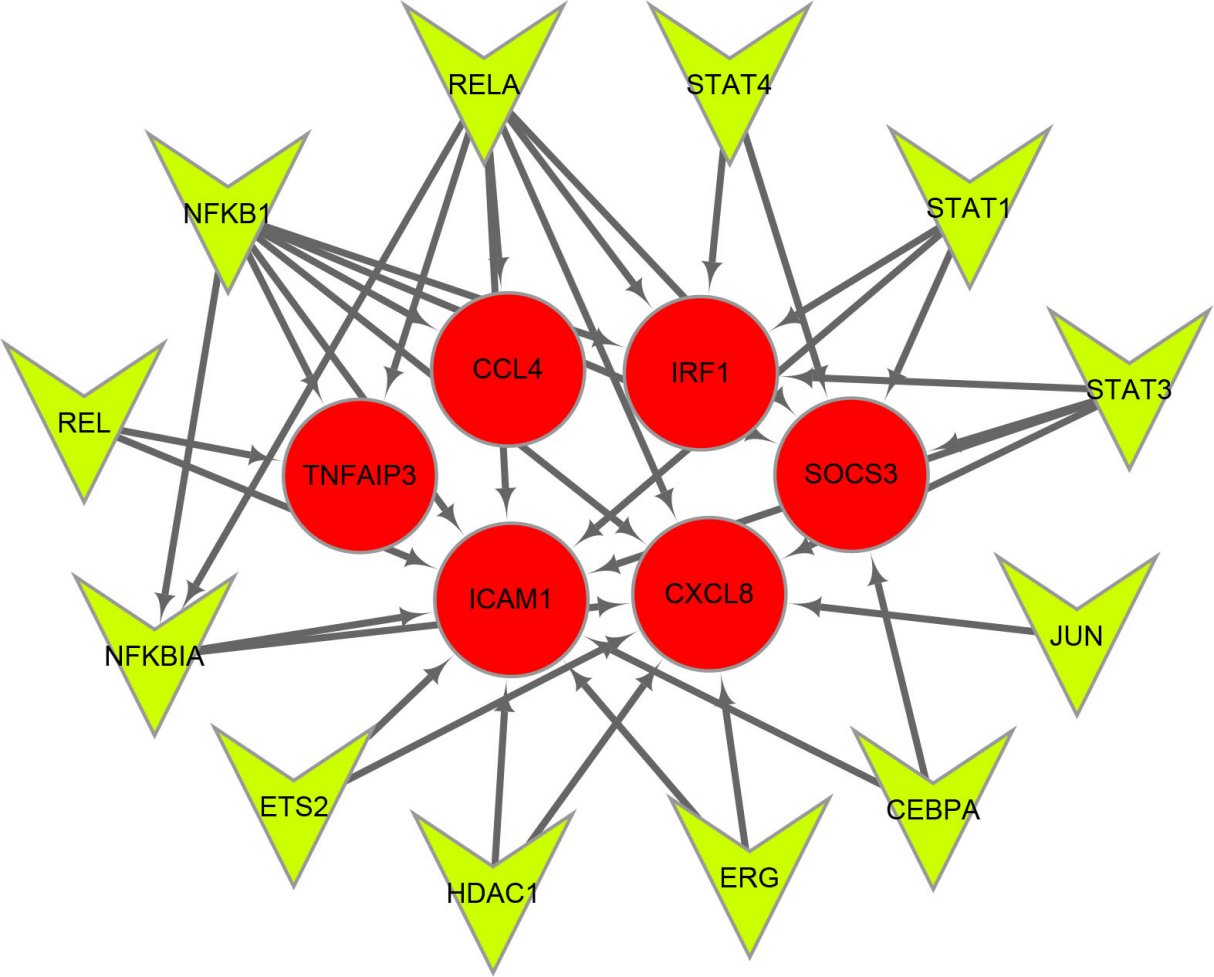
Transcription factors (TFs) regulatory network of immune-related hub genes. TFs are represented as green triangles and immune-related hub genes are represented as red circles.

Twelve TFs were found to regulate these immune-related hub genes based on the TRRUST database (**Figure 6**). Detailed information of the 12 TFs is shown in **Table 3**. At the same time, we found that the Hub genes JUN and NFKBIA are also upstream transcription factors regulating these Hub genes. NFKBIA may be regulated by the transcription factors, NFKB1 and RELA, and may also act as a transcription factor that regulates ICAM1 and CXCL8. JUN acts as a transcription factor that regulates CXCL8.

**Table 3:** Key transcriptional factors (TFs) of hub genes.

Key TFs	Description	P-value	Target Genes
RELA	v-rel reticuloendotheliosis viral oncogene homolog A (avian)	8.56E-12	CCL4, NFKBIA, CXCL8, ICAM1, TNFAIP3, SOCS3, IRF1
NFKB1	nuclear factor of kappa light polypeptide gene enhancer in B-cells 1	8.97E-12	TNFAIP3, IRF1, SOCS3, CXCL8, ICAM1, NFKBIA, CCL4
STAT3	signal transducer and activator of transcription 3 (acute-phase response factor)	3.76E-07	aIRF1, ICAM1, SOCS3, CXCL8
STAT1	signal transducer and activator of transcription 1, 91kDa	7.01E-06	IRF1, ICAM1, SOCS3
STAT4	signal transducer and activator of transcription 4	1.11E-05	SOCS3, IRF1
NFKBIA	nuclear factor of kappa light polypeptide gene enhancer in B-cells inhibitor, alpha	3.44E-05	ICAM1, CXCL8
REL	v-rel reticuloendotheliosis viral oncogene homolog (avian)	4.65E-05	ICAM1, TNFAIP3
ERG	v-ets erythroblastosis virus E26 oncogene homolog (avian)	7.05E-05	ICAM1, CXCL8
ETS2	v-ets erythroblastosis virus E26 oncogene homolog 2 (avian)	9.96E-05	CXCL8, ICAM1
CEBPA	CCAAT/enhancer binding protein (C/EBP), alpha	0.000255	ICAM1, SOCS3`
HDAC1	histone deacetylase 1	0.000494	CXCL8, ICAM1
JUN	jun proto-oncogene	0.00215	CXCL8, JUN

### Further mining of MiRNA and prediction of LncRNA

**Figure 7:**
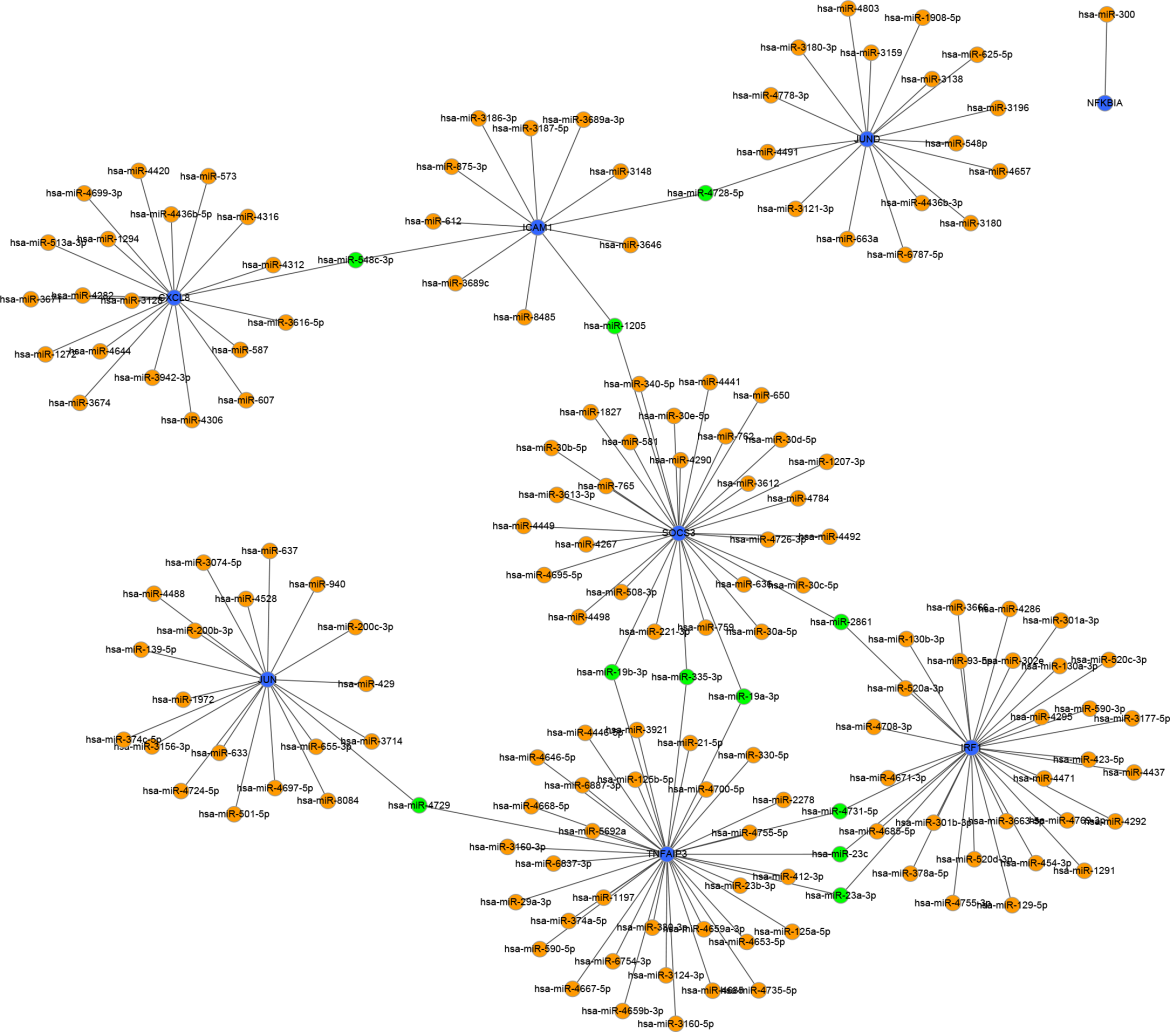
Interaction network between immune-related hub genes and their targeted miRNAs. The immune-related hub genes are represented as blue, the miRNAs are represented as orange, and the cross-linked miRNAs are represented as green.

**Figure 8:**
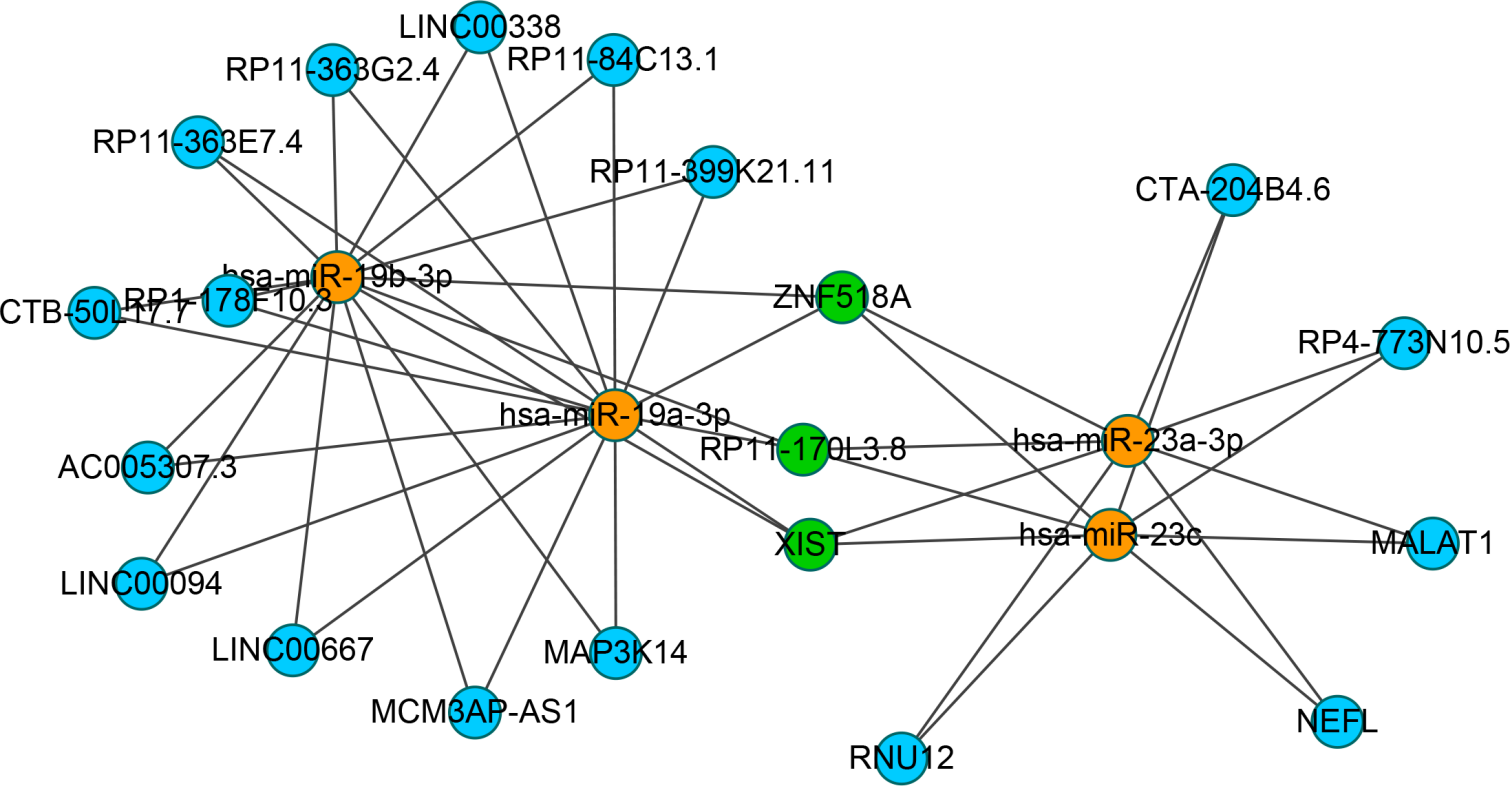
Interaction network between cross-linked miRNAs and their targeted LncRNAs. The miRNAs are represented as organge, the LncRNAs are represented as blue, and the cross-linked LncRNAs are represented as green.

To identify the upstream miRNAs of these hub genes and ensure the accuracy and reliability of the results, we took intersections of the miRNAs of these genes predicted by each of the three databases. A total of 161 predicted upstream miRNAs were obtained, of which no targeted miRNAs were identified for CCL4 (**Figure 7**). The miRNAs with a high number of cross-linked genes (≥2) are shown in **Table 4**. Eleven miRNAs had a higher number of cross-linked genes (≥2), of which only hsa-miR-19b-3p, hsa-miR-19a-3p, has-miR-23a-3p, has-miR-23c had the upstream molecule LncRNA and had higher reliability, all targeting TNFAIP3. The lncRNAs corresponding to hsa-miR-19b-3p, hsa-miR-19a-3p, has-miR-23a-3p, has-miR-23c were predicted using StarBase 2.0, and the highest reliability (very high stringency, >5) was selected as the criterion. After cross-linking the lncRNAs and miRNAs, three lncRNAs targeting four key miRNAs, ZNF518A, RP11-170L3.8, and XIST, were identified (**Figure 8**).

**Table 4:** miRNAs and its target genes.

miRNA	Genes targeted by miRNA	Gene count
has-miR-54c-3p	CXCL8, ICAM1	2
has-miR-4728-5p	ICAM1, JUND	2
has-miR-1205	ICAM1, SOCS3	2
has-miR-19b-3p	SOCS3, TNFAIP3	2
has-miR-335-3p	SOCS3, TNFAIP3	2
has-miR-19a-3p	SOCS3, TNFAIP3	2
has-miR-4729	JUN, TNFAIP3	2
has-miR-2861	SOCS3, IRF1	2
has-miR-4731-5p	IRF1, TNFAIP3	2
has-miR-23c	IRF1, TNFAIP3	2
has-miR-23a-3p	IRF1,TNFAIP3	2

### Validation of vascular clamping-induced I/R injury mouse model and hub genes expression

**Figure 9:**
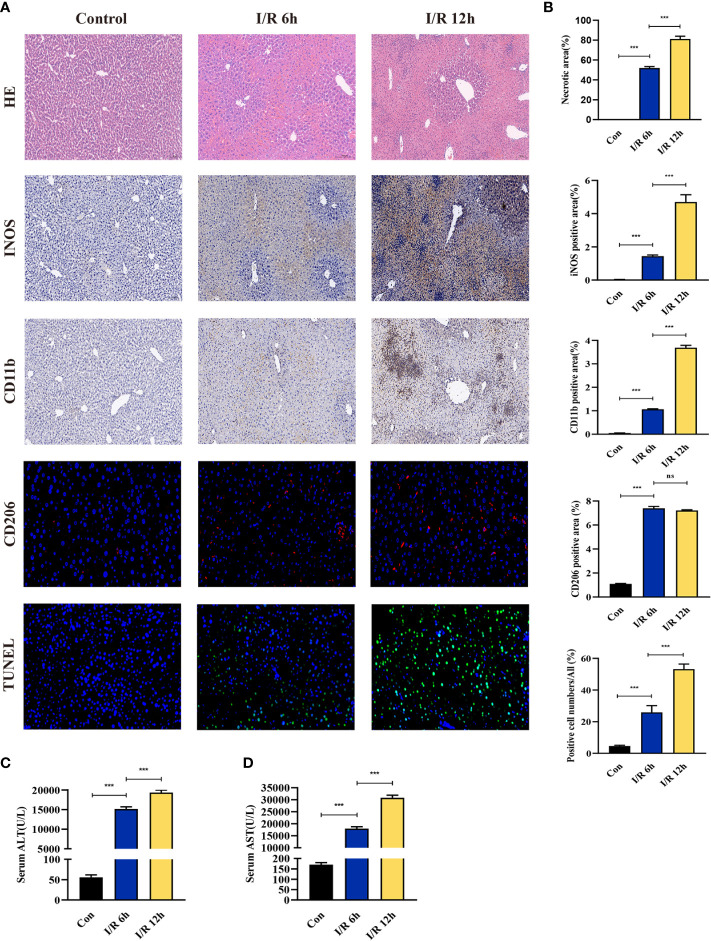
Evaluation of liver injury, hepatocyte apoptosis, and immune cell infiltration in a hepatic ischemia-reperfusion induced injury mouse model. **(A)** Representative pictures of HE staining, immunohistochemical staining, immunofluorescence staining, and TUNEL staining (magnification, 40x). **(B)** Quantification of liver injury score, immune cell-related molecules in the INOS, CD11b, and CD206 positive areas, and apoptosis of liver cells. **(C, D)** Serum ALT/AST activity at different reperfusion times after hepatic ischemia. ***p < 0.0001. ns=non-significant.

**Figure 10:**
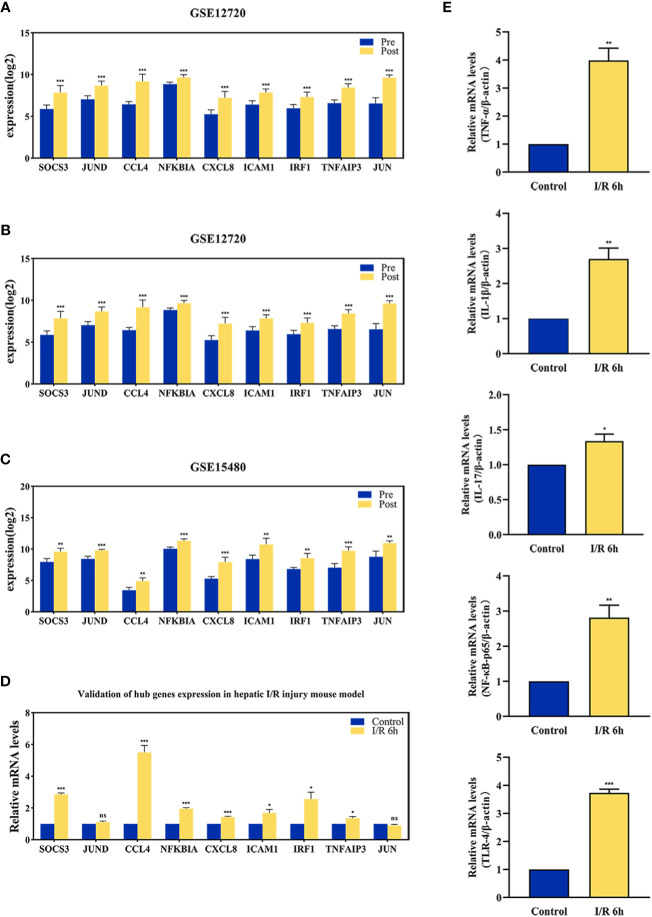
The validation of immune-related hub genes expression and innate immune signaling. The expression level of hub genes in GSE12720 **(A)**, GSE14951 **(B)**, and GSE15480 **(C)**. **(D)** The expression level of hub genes in mouse at 6 h after I/R or sham group. **(E)** mRNA levels of TNF-α, IL-1β, IL-17, NF-κB-p65, and TLR-4 in mouse at 6 h after I/R or sham group. *p < 0.01; **p < 0.001; ***p < 0.0001. ns=non-significant.

The effect of hepatic I/R injury after clamping of blood vessels was verified, and the extent of apoptosis and associated immune cell infiltration was assessed. After experiencing 1 hour of ischemia, the left and middle lobe livers of mice were reperfused. As the early reperfusion time was lengthened, HE staining of the livers of mice revealed increasingly severe liver necrosis (12h>6h). Hepatocyte apoptosis and levels of the blood biochemical indexs, ALT and AST, also increased with longer reperfusion times (**Figures 9A–D**). Immunohistochemical staining analysis of the macrophage-associated molecule INOS (M1) and the neutrophil-associated molecule CD11b showed a significant increase in M1 macrophage and neutrophil infiltration at longer reperfusion times. The immunofluorescence staining analysis of the macrophage-associated molecule CD206 (M2) also showed a obvious increase in M2 macrophage infiltration after reperfusion (**Figures 9A, B**). Expression of these immune-related hub genes was validated in the three databases, indicating that they were all significantly upregulated after hepatic I/R injury(**Figures 10A–C**). A mouse model of hepatic I/R injury was used to verify the expression levels of immune-related hub genes by qRT-PCR. As shown in **Figure 10D**, the expression levels of SOCS3, CCL4, NFKBIA, CXCL8, ICAM1, IRF1, and TNFAIP3 were significantly upregulated after 6 hours of I/R, consistent with the findings of GEO datasets. In contrast, the expression levels of JUN and JUND did not differ, which may be related to species, reperfusion time. A number of immune signalling downstream molecules were validated and TNF-α, IL-1β, IL-17, NF-κB-p65 and TLR-4 were significantly upregulated after 6h of hepatic ischemia-reperfusion, further confirming the involvement of innate immune signaling identified in the study in the process of hepatic ischemia-reperfusion injury (**Figure 10E**).

## Discussion

Liver transplantation is the only effective treatment for end-stage liver disease. However, the number of donor livers is far from adequate, requiring clinicians to consider marginal or expanded criteria livers, such as those donated after circulatory death (DCD) (20). Early allograft dysfunction (EAD) is an important cause of morbidity and mortality in liver transplant recipients, while I/R injury is a major cause of EAD and a major risk factor for acute and chronic rejection after transplantation, and is a contributing factor to the shortage of organs available for transplantation (21, 22). These adverse effects are a more serious problem in the expanded standard transplantation liver. Thus, better understanding the mechanisms of hepatic I/R injury is needed to develop new strategies for improving clinical outcomes and expanding the donor pool. Analysis of gene expression profiles based on liver transplantation may help to define the pathogenesis of hepatic I/R injury. The current study shows for the first time the key molecules and mechanisms involed in the liver transplantation process, and makes upstream predictions about these core molecules.

There are two main stages of hepatic I/R injury. The first stage is ischemia injury caused by inadequate blood supply while the second stage is reperfusion injury caused by immune activation and overproduction of reactive oxygen species (ROS) (23). Injury induced by reperfusion is a key factor affecting patient prognosis, induced mainly by ROS and severe inflammatory immune responses involving direct and indirect cytotoxic mechanisms (5). The main processes include the activation of innate immune cells, secretion of inflammatory cytokines and chemokines, production of ROS, elevated expression of adhesion molecules and infiltration of circulating immune cells (24). The immune response is usually induced by ischemia-induced hepatocyte death, which can release damage-associated molecular patterns (DAMPs) that activate inflammation (25). Thus, there is a clear need to understand the immune-related mechanisms associated with hepatic I/R injury.

In this study, we identified immune-related hub genes involved in hepatic I/R injury and further explored the associated pathogenesis. A total of 71 DEGs from the intersection of DEGs identified in the GSE12720, GSE14951 and GSE15480 datasets were obtained. Pathway enrichment analysis of the DEGs were performed using Rand these genes were found to be primarily enriched in immune and inflammation-related pathways. Modular genes obtained from MCODE were then analyzed and genes significantly enriched in the IL-17 signaling pathway, including FOSB, JUN, TNFAIP3, JUND, CXCL8, NFKBIA, FOSL1, and CEBPB, were identified. To investigate the role of immune-related genes in hepatic I/R injury, we took intersections of the top 20 genes obtained using the cytoHubba plug-in of Cytoscape and immune-related genes, and eventually obtained nine immune-related hub genes, including SOCS3, JUND, CCL4, NFKBIA, CXCL8, ICAM1, IRF1, TNFAIP3, and JUN. These key genes play a synergistic role in mediating lipopolysaccharide responses, chemokines, leukocyte migration and cytokine activity. In addition, twelve TFs were found to regulate the expression of these genes, with NFKBIA and JUN again acting as TFs to regulate these hub genes. These findings illustrate that bioinformatics can aid the some prediction of the molecular mechanisms of immunity associated with hepatic I/R injury.

SOCS3, a member of the SOCS family, is an endogenous regulator of the Janus kinase (JAK)/signal transducer and activators of transcription (STAT) pathway. SOCS3 can negatively regulate STAT3 (26), suppressing the M1 pro-inflammatory phenotype, and inactivating the inflammatory response in macrophages (27). However, studies indicate that downregulation of SOCS3 enhances IL6/STAT3 expression to promote hepatocyte proliferation during liver injury, resection and transplantation (28). The JUN family consists of c-Jun, JunB and JunD, of which c-JUN and JUND are significantly upregulated following hepatic I/R (29). The DNA-binding activity of activated protein-1 (AP-1) is significantly increased after hepatic I/R and predominately consists of c-JUN and JUND hetero- and homodimers (30). Marden et al. found that JUND inhibits AP-1-mediated transcriptional activation of cell cycle regulatory genes after hepatic I/R injury, allowing time for the liver to repair cellular damage before proliferation (31). In contrast, Wu et al. showed that JUND expression exacerbates I/R injury in the liver (32). JUND exhibits different functions, likely due to the temporal sequence after reperfusion. c-JUN plays an important role in hepatocyte proliferation and survival during liver development and regeneration (33). I/R injury can also activate apoptosis in hepatocytes by increasing c-JUN expression (34).

CCL4 belongs to the macrophage inflammatory protein-1 (MIP-1) family, which recruits immune cells and is produced by hepatic stellate cells, macrophages and monocytes, in the liver (35). Krohn et al. found that CCL4 plays an important role in the early induction of immune cell infiltration after hepatocyte transplantation (36). NFKBIA, also known as IKB-α, is a member of the NF-Kappa-B inhibitor family. Suetsugu et al. used an IKBα super-repressor to inactivate NF-KB and effectively blocked short-term hepatic warm I/R injury (37). NFKBIA is also shown to play a role in myocardial and cerebral I/R injury (38), a finding supporting the current study.

CXCL8, also known as IL-8, activates chemokine receptors to induce inflammation (39). Lv et al. found that the activation of glial cells and suppression of neuroinflammation can be promoted during ischemic stroke by inhibiting CXCL8 (40). The current study also showed an upregulation of CXCL8, while some studies have confirmed that CXCL8 increases with ischemia and rejection after liver transplantation (41). ICAM-1 is a cell adhesion factor that promotes the recruitment of leukocytes to sites of inflammation (42). Farhood et al. found that ICAM-1 plays an important role in the neutrophil-dependent phase of injury after hepatic ischemia and reperfusion (43). Thus, blocking this adhesion molecule is potentially valuable for the treatment of acute liver failure following ischemia. IRF1 is a transcription factor that regulates gene expression during immunization, and studies have shown that IRF1 promotes I/R injury during liver transplantation (44). Yang et al. found that IRF-1 regulates Rab27a transcription and extracellular vesicle secretion, leading to oxidative phospholipid activation in neutrophils and subsequent hepatic I/R injury (45). Meanwhile, Yokota et al. found that IRF1 exacerbates liver transplant I/R injury by promoting IL-15/IL-15Rα production by hepatocytes (46). TNFAIP3, or A20, is a negative regulator of NF-KB. While the inhibition of NF-KB by TNFAIP3 can exacerbate hepatic I/R injury (47), TNFAIP3 knockdown increases the infiltration of inflammatory cells into the liver (48). Given the different roles of TNFAIP3 in the regulation of hepatic I/R injury and liver immunity, further in-depth studies on the role of TNFAIP3 in regulating the immune process of liver I/R injury are needed in the future.

The current study evaluated liver damage at different I/R times. As reperfusion time increased during the initial phase of disease, hepatic damage become more severe and neutrophil and M1 macrophage infiltration gradually increased. These findings suggesting immune cells play an important role in regulating the early stages of liver damage. This study also had several limitations. First, while three microarray datasets of human liver transplantation patients were analyzed, the sample size remained small. There may be ethical and other reasons why it is difficult to obtain a post-transplant sample of human liver. Second, no miRNA, LncRNA and TFs microarray datasets of patients with liver transplantation was available from an open database, so potential targets could only be predicted using by online tools. In conclusion, few studies have explored I/R injury in the liver using a bioinformatics approach. This study used three databases to explore the hub molecular mechanisms of immune-related I/R injury in liver transplantation and to predict their regulatory non-coding RNAs and transcription factors. The findings provide new insights into the pathogenesis of hepatic I/R injury and potential therapeutic targets.

## Data availability statement

Publicly available datasets were analyzed in this study. This data can be found here: immune-related gene (http://www.immport.org/) and the Gene expression Omnibus (GEO) database (https://www.ncbi.nlm.nih.gov/geo/) (Accession: GSE12720, GSE14951 and GSE15480).

## Ethics statement

The animal study was reviewed and approved by the Laboratory Animal Welfare and Ethics Committee of the Renmin Hospital of Wuhan University.

## Author contributions

TQ and JZ designed the study. JG and SH analyzed data, drawn charts. JG and QC wrote manuscripts. TW and BY helped collect data and references. JG, SH, QC, TW, BY performed the experiments. All authors contributed to the article and approved the submitted version.
