# Supplementary material for: Analysis of potential immune-related genes involved in the pathogenesis of ischemia-reperfusion injury following liver transplantation
Source: Front Immunol. 2023 Mar 16;14:1126497. doi: 10.3389/fimmu.2023.1126497 (PMC10060527; doi:10.3389/fimmu.2023.1126497)
Supplement: Supplementary file 1 [file Table_1.doc]

**Table S1 Sequence of primers used for qRT-PCR.**

| **Gene** |  | **Sequence (5’ to 3’)** |
| --- | --- | --- |
| M-Jun | Forward | CACCACTTGCCCCAACAGAT |
|  | Reverse | TTCCTCATGCGCTTCCTCTCT |
| M-Nfkbia | Forward | CACTTGGTGACTTTGGGTGCT |
|  | Reverse | GCTGTATCCGGGTACTTGGG |
| M-Cxcl8 | Forward | GGCATCTTCGTCCGTCCCT |
|  | Reverse | CCAACAGTAGCCTTCACCCAT |
| M-Icam1 | Forward | GTACTGTACCACTCTCAAAATAACTGG |
|  | Reverse | TGGGGCTTGTCCCTTGAGT |
| M-Socs3 | Forward | GCGGGCACCTTTCTTATCC |
|  | Reverse | TGGATGCGTAGGTTCTTGGTC |
| M-Tnfaip3 | Forward | TGGGATTTATCTGCCTCTTCAC |
|  | Reverse | CACAGGGATCTCCATCACTATCAAG |
| M-Irf1 | Forward | CAGGAACCAGAGGAAAGAGAGAA |
|  | Reverse | GCTGTGGTCATCAGGTAGGGTAG |
| M-Ccl4 | Forward | CCCAGCTCTGTGCAAACCTA |
|  | Reverse | CCATTGGTGCTGAGAACCCT |
| M-Jund | Forward | GACCAGTACGCAGTTCCTCTACC |
|  | Reverse | AACTGCTCAGGTTGGCGTAGA |
| M-Tnf-α | Forward | CCCTCACACTCACAAACCACC |
|  | Reverse | CTTTGAGATCCATGCCGTTG |
| M-Tlr4 | Forward | TGAGGACTGGGTGAGAAATGAGC |
|  | Reverse | CTGCCATGTTTGAGCAATCTCAT |
| M-Il-1β | Forward | GCATCCAGCTTCAAATCTCGC |
|  | Reverse | TGTTCATCTCGGAGCCTGTAGTG |
| M-Il-17 | Forward | TCCACCGCAATGAAGACCCT |
|  | Reverse | CATGTGGTGGTCCAGCTTTCC |
| M-Nf-κB-p65 | Forward | CGAGTCTCCATGCAGCTACG |
|  | Reverse | TTTCGGGTAGGCACAGCAATA |
| M-β-actin | Forward | GTGACGTTGACATCCGTAAAGA |
|  | Reverse | GTAACAGTCCGCCTAGAAGCAC |
